# Large-scale development of cost-effective SNP marker assays for diversity assessment and genetic mapping in chickpea and comparative mapping in legumes

**DOI:** 10.1111/j.1467-7652.2012.00710.x

**Published:** 2012-08

**Authors:** Pavana J Hiremath, Ashish Kumar, Ramachandra Varma Penmetsa, Andrew Farmer, Jessica A Schlueter, Siva K Chamarthi, Adam M Whaley, Noelia Carrasquilla-Garcia, Pooran M Gaur, Hari D Upadhyaya, Polavarapu B Kavi Kishor, Trushar M Shah, Douglas R Cook, Rajeev K Varshney

**Affiliations:** 1 International Crops Research Institute for the Semi-Arid Tropics (ICRISAT)Patancheru, India; 2 Osmania UniversityHyderabad, India; 3 University of CaliforniaDavis, CA, USA; 4 National Center for Genome Resources (NCGR)Santa Fe, NM, USA; 5 University of North CarolinaCharlotte, NC, USA; 6 Generation Challenge Program (GCP)Mexico DF, Mexico

**Keywords:** chickpea, single nucleotide polymorphisms, KASPar assay, linkage map, legume synteny

## Abstract

A set of 2486 single nucleotide polymorphisms (SNPs) were compiled in chickpea using four approaches, namely (i) Solexa/Illumina sequencing (1409), (ii) amplicon sequencing of tentative orthologous genes (TOGs) (604), (iii) mining of expressed sequence tags (ESTs) (286) and (iv) sequencing of candidate genes (187). Conversion of these SNPs to the cost-effective and flexible throughput Competitive Allele Specific PCR (KASPar) assays generated successful assays for 2005 SNPs. These marker assays have been designated as Chickpea KASPar Assay Markers (CKAMs). Screening of 70 genotypes including 58 diverse chickpea accessions and 12 BC_3_F_2_ lines showed 1341 CKAMs as being polymorphic. Genetic analysis of these data clustered chickpea accessions based on geographical origin. Genotyping data generated for 671 CKAMs on the reference mapping population (*Cicer arietinum* ICC 4958 × *Cicer reticulatum* PI 489777) were compiled with 317 unpublished TOG-SNPs and 396 published markers for developing the genetic map. As a result, a second-generation genetic map comprising 1328 marker loci including novel 625 CKAMs, 314 TOG-SNPs and 389 published marker loci with an average inter-marker distance of 0.59 cM was constructed. Detailed analyses of 1064 mapped loci of this second-generation chickpea genetic map showed a higher degree of synteny with genome of *Medicago truncatula*, followed by *Glycine max*, *Lotus japonicus* and least with *Vigna unguiculata*. Development of these cost-effective CKAMs for SNP genotyping will be useful not only for genetics research and breeding applications in chickpea, but also for utilizing genome information from other sequenced or model legumes.

## Introduction

Chickpea (*Cicer arietinum*) is the third most important legume crop, a source of dietary protein and a beneficial agricultural crop in the semi-arid regions of the world. The development of sustainable high yielding varieties against persisting abiotic stresses and biotic stresses is a prerequisite to meet the world hunger. Molecular breeding strategies have been adopted to improvise crop improvement programmes in several crops including legumes such as soybean and common bean (see Chamarthi *et al.*, 2011). In case of chickpea, progress in the area of implementation of markers in breeding programmes, however, has been relatively slow. Availability of limited molecular markers coupled with narrow genetic diversity has been the major constraints to hamper development of genetic maps and undertaking trait mapping studies. Marker genotyping cost is another critical factor that determines adoption of markers in breeding programmes as it involves genotyping of large number of segregating lines.

Among different marker systems, simple sequence repeats (SSRs) and SNPs are the markers of choice for genetics and plant breeding applications (Close *et al.*, 2009; Gupta and Varshney, 2000). Although the genotyping assays are expensive and/or time consuming, the SSR markers have been an inevitable choice till date in many crop species including chickpea for large-scale characterization of germplasm collections (Upadhyaya *et al.*, 2008), construction of genetic maps (Choudhary *et al.*, 2009; Nayak *et al.*, 2010; Thudi *et al.*, 2011; Winter *et al.*, 1999) and QTL identification (Aryamanesh *et al.*, 2010; Santra *et al.*, 2000). On the other hand, SNPs are biallelic and the most abundant genetic variations, which are evenly distributed in higher frequencies throughout the genome of most plant species (Allen *et al.*, 2011; Yan *et al.*, 2009). As these markers are amenable for automation and high-throughput approach, the genotyping costs for SNPs can be lowered down. As a result, SNP genotyping of large-scale segregating populations as well as germplasm collections becomes cost-effective for developing high-density genetic maps, genome-wide association mapping, marker-assisted selection (MAS) and genomic selection (GS) studies (see Varshney, 2010).

Depending on the sample size and number of markers to be analysed, medium- to high-throughput assay platforms such as BeadXpress and GoldenGate assays from Illumina Inc. (San Diego, CA) with varying set of multiplexes (96, 384, 768 or 1536 SNPs per assay) are available. Such platforms have been developed and used in several crop species such as barley (Close *et al.*, 2009), wheat (Akhunov *et al.*, 2009), maize (Yan *et al.*, 2009), oil seed rape (Durstewitz *et al.*, 2010), soybean (Hyten *et al.*, 2008), cowpea (Muchero *et al.*, 2009), pea (Deulvot *et al.*, 2010) and chickpea (Choudhary *et al.*, 2012; R.V. Penmetsa, N. Carraquilla-Garcia, A.D. Farmer, R.K. Varshney, D.R. Cook, unpublished data). These platforms, however, are cost-effective only when a minimum of 96, 384, 762 or 1536 SNPs are used for genotyping a large number of genotypes (R.R. Mir, P.J. Hiremath, O. Riera-Lizarazu, R.K. Varshney, unpublished results). In cases of molecular breeding applications such as MAS where only few markers are required for genotyping a large number of segregating lines, Illumina-based genotyping assays do not seem to be cost-effective. In such cases, Competitive Allele Specific PCR (KASPar) assay from KBiosciences (Hertfordshire, UK) (http://www.kbioscience.co.uk) seems to be an attractive marker genotyping assay (Allen *et al.*, 2011; Cortes *et al.*, 2011). KASPar assay is a PCR-based novel homogeneous fluorescent SNP genotyping system. It is a very flexible assay and can be carried out on undefined set of markers (http://www.kbioscience.co.uk/reagents/KASP_manual.pdf, http://www.kbioscience.co.uk/download/KASP.swf).

This study has been undertaken in chickpea with the following objectives: (i) to compile a large set of informative SNPs, (ii) to develop KASPar assays for cost-effective SNP genotyping, (iii) to analyse genetic diversity in the selected *Cicer* spp. accessions, (iv) to develop a second-generation genetic map based on SNPs, and (v) to determine the extent of genetic synteny of chickpea with some closely related legume species.

## Results

### Large-scale identification of SNPs

**Figure 1:**
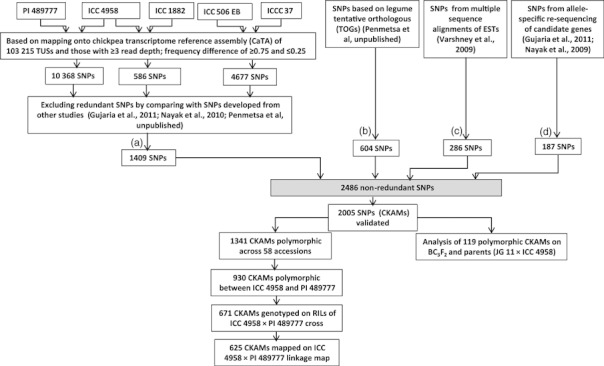
A schematic representation to select the informative SNPs for conversion into KASPar assay and their utilization for genetic mapping and germplasm analysis. A total of four approaches—(i) Solexa/Illumina sequencing, (ii) tentative orthologous genes (TOGs), (iii) mining of expressed sequence tags from public domain (iv) and allele-specific resequencing—were used to identify a set of 2486 nonredundant SNPs. Although efforts were made to develop KASPar assays for all SNPs, successful assays were developed for 2005 SNPs. Screening of these assays on 58 *Cicer* spp. accessions showed polymorphism with 1341 CKAMs, including 119 CKAMs showed polymorphism with JG 11 and ICC 4958, the parental lines of 12 BC_3_F_2_ lines analysed. Furthermore, genotyping data were generated for 651 CKAMs on 131 RILs of the interspecific mapping populations, of which 625 CKAMs were integrated into the chickpea genetic map.

With an objective of developing the cost-effective KASPar assays for chickpea genetics and breeding applications, 2486 informative SNPs were compiled following four approaches (Figure 1).

#### Solexa/Illumina sequencing

Solexa/Illumina 1G sequencing was carried out on total RNA samples of four genotypes, namely ICC 4958, ICC 1882, ICC 506-EB and ICCC 37 of the cultivated species (*C. arietinum*), and one genotype (PI 489777) of wild species (*Cicer reticulatum*) (Hiremath *et al.*, 2011). In total, approximately 96 million Solexa/Illumina sequence reads were generated (Table 1). After aligning these sequence reads with the chickpea transcriptome assembly (CaTA) comprising 103 215 tentative unique sequences (TUSs) (Hiremath *et al.*, 2011) using Alpheus pipeline (Miller *et al.*, 2008) and pair-wise comparison of parental genotypes considering selection criteria such as read depth of ≥3 and frequency difference of ≥0.75 and ≤0.25 (Azam *et al.*, 2012), a total of 15 361 SNPs in 9517 TUSs were selected (Table 1). By comparing the identified SNPs across the three parental combinations, 14 454 unique SNPs were identified from 9517 nonredundant TUSs. To select nonredundant SNPs, all the 14 454 SNPs in 9517 TUSs were compared with already available SNPs developed in other studies (Gujaria *et al.*, 2011; Nayak *et al.*, 2010; R.V. Penmetsa, N. Carraquilla-Garcia, A.D. Farmer, R.K. Varshney, D.R. Cook, unpublished results). As a result, a final set of 1409 SNPs from 1409 TUSs was selected.

**Table 1:** A summary of identification of single nucleotide polymorphisms (SNPs) based on Solexa/Illumina sequencing

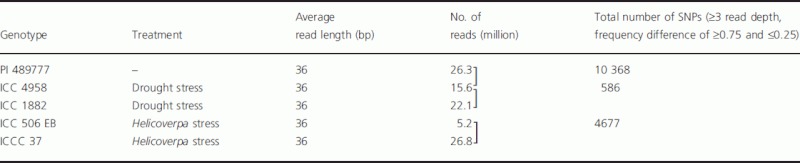

#### Mining of sanger ESTs

On the basis of cluster analysis of 27 259 Sanger expressed sequence tags (ESTs), 9569 unigenes including 2431 contigs and 7138 singletons were identified in an earlier study (Varshney *et al.*, 2009). A set of 729 contigs having ESTs from at least two genotypes and read depth of ≥5 was explored for SNP selection. An SNP with high polymorphism information content (PIC) value (≥0.5) and having at least 50 bp window on either sides was considered from each contig. Finally, a nonredundant set of 286 SNPs from 286 TUSs were selected (Figure 1).

#### Allele-specific sequencing of candidate genes

Allele resequencing of 220 genes on a set of 2–20 genotypes representing nine *Cicer* species provided 1893 SNPs in our earlier study (Gujaria *et al.*, 2011). By considering the criteria of selecting one SNP with higher PIC value from each gene and 50-bp region on both flanking side of the SNP, a total of 183 SNPs present in 183 genes were selected. In addition, four SNPs coming from two drought-responsive genes (Nayak *et al.*, 2009) were also selected (Figure 1).

#### Allele-specific sequencing of TOGs

With a goal of identification of cross-species genetic markers, allele-sequencing was conducted on ICC 4958 and PI 489777 for a total of 1440 tentative orthologous genes (TOGs) (R.V. Penmetsa, N. Carraquilla-Garcia, A.D. Farmer, R.K. Varshney, D.R. Cook, unpublished data). On the basis of SNP analysis on this data set, a GoldenGate assay was developed for 768 SNPs including 733 SNPs from TOGs and 155 SNPs from other sources. Genotyping of the reference mapping population with this GoldenGate assay integrated a total of 450 SNPs including 429 TOG-SNPs onto the genetic map. On the basis of designable criteria for KASPar assays, a total of 604 TOG-SNPs including 410 mapped and 194 unmapped SNPs were selected (Figure 1).

In brief, a set of 2486 SNPs including 1409 SNPs from Solexa/Illumina sequencing, 286 SNPs from mining Sanger ESTs, 187 SNPs from allele-specific sequencing of candidate genes and 604 TOG-SNPs was assembled (Table S1). It is important to mention here that except for the 187 SNPs from allele resequencing of candidate genes and 604 SNPs from TOGs, the assembled SNPs were not validated earlier. Therefore, the compiled SNPs can be considered as putative SNPs.

### Development and validation of KASPar assay

The selected set of 2486 SNPs was used for developing KASPar assays (Table S1). The developed KASPar assays have been designated as Chickpea KASPar Assay Markers (CKAMs). All 2486 CKAMs were used for validation on a panel of 70 genotypes (Table S2). These genotypes include 55 lines/varieties of the cultivated species (*C. arietinum*) from 11 countries, three accessions from the wild species (*C. reticulatum*) and 12 BC_3_F_2_ lines generated after introgressing a genomic region containing QTLs for several drought tolerance traits from ICC 4958 into JG 11 by using marker-assisted backcrossing approach (unpublished results).

**Figure 2:**
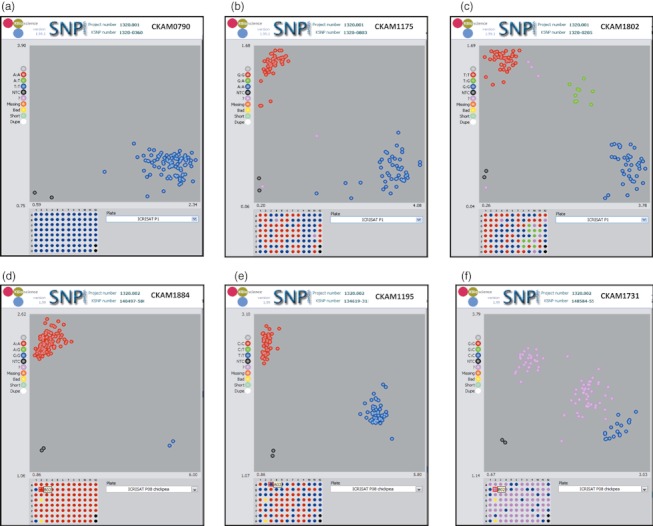
Snapshots showing SNP genotyping with KASPar assays. Different possible scenarios of SNP genotyping in germplasm collection (a–c) and interspecific RIL mapping population (d–f) have been shown. Marker genotyping data generated for each genotype were used for allele calling using the automatic allele calling option. Allelic discrimination (two alleles) for a particular marker in the genotypes examined has been shown on a scatter plot with axes ‘*X*’ and ‘*Y*’. The snapshot (a) shows monomorphic pattern, that is, occurence of only one allele (blue spots) for CKAM0790 marker. In the snapshot (b), polymorphism pattern, that is, occurence of two alleles (blue and red spots) for CKAM1175 marker in almost equal proportion in the germplasm collection, has been shown. All germplasm accessions show homozygosity for the corresponding alleles, and one accession shows missing data (pink spot). The snapshot (c) shows heterozygosity, that is, occurence of both alleles (green spots) for CKAM1802 marker in nine germplasm accessions in addition to occurence of two alleles in homozygous condition in several accessions (blue and red spots) and three accessions with missing data. The snapshot (d) shows occurence of one allele (red spots) in majority of RILs, except two RILs with the other allele (blue spots) and two RILs with missing data (brown spots). Two clusters of about 50% of RILs each with one allele (blue and red spots) along with two RILs with missing data (brown spots) have been shown in the snapshot (e). The snapshot (f) shows occurence of one allele (blue spots) in several RILs and missing data in majority of the lines.

A total of 2005 (80.6%) CKAMs were validated of the 2486; of these, 1341 (66.8%) CKAMs were polymorphic among 58 genotypes, 664 (33.1%) were monomorphic in the genotypes tested, and 481 (19.4%) failed to generate a useful amplification signal (Table S1, Figure 2). No attempt was made to redesign the primer for failed CKAMs. A comparison of SNP predicted *in silico* (assembled) and alleles called in the KASPar assays for the 2005 validated CKAMs showed 100% consistency. The PIC values for the polymorphic CKAMs varied between 0.02 and 0.50 with an average of 0.12 (Table S1).

Analysis of CKAMs on the parental genotypes of the mapping populations showed higher polymorphisms in interspecific (*C. arietinum* × *C. reticulatum*) crosses than in intraspecific (*C. arietinum* × *C. arietinum*) crosses. Among interspecific crosses, maximum number of polymorphisms (930 CKAMs) was observed in the reference mapping population (ICC 4958 × PI 489777) followed by crosses segregating for *Helicoverpa* resistance, that is, ICC 3137 × IG 72953 (620 CKAMs) and ICC 3137 × IG 72933 (276 CKAMs). In the case of the intraspecific crosses, maximum polymorphism was identified between Arerti and ICC 4958 (159 CKAMs), which represent parents of MABC population for improvement of chickpea for drought tolerance. The polymorphism status of CKAMs between different parental combinations is given in Table 2.

**Table 2:** CKAMs-based polymorphisms in some segregating populations of chickpea

Parental genotypes of segregating population	Features of segregating populations	Marker data available for both parental lines	Polymorphic markers (%)
Interspecific mapping populations (*Cicer arietinum* × *Cicer reticulatum*)			
ICC 4958 × PI 489777	International reference mapping population	1900	930 (48.9)
ICC 3137 × IG 72953	*Helicoverpa* resistance	1744	620 (35.6)
ICC 3137 × IG 72933	*Helicoverpa* resistance	1839	276 (15.0)
Intraspecific mapping populations (*C. arietinum* × *C. arietinum*)			
ICC 4958 × ICC 1882	Drought tolerance and root traits	1966	148 (7.5)
ICC 283 × ICC 8261	Drought tolerance and root traits	1960	58 (3.0)
ICC 6263 × ICC 1431	Salinity tolerance	1966	54 (2.7)
JG 62 × ICCV 05530	*Fusarium* wilt (FW), *Ascochyta* blight (AB), *Botrytis* grey mould (BGM)	1947	32 (1.6)
Annigeri × ICC 4958	Root traits	1939	125 (6.4)
ICC 506-EB × Vijay	*Helicoverpa* resistance	1969	27 (1.4)
Marker-assisted backcrossing (MABC) populations			
Arerti × ICC 4958	Introgressing root trait QTL	1964	159 (8.1)
Ejere × ICC 4958	Introgressing root trait QTL	1967	140 (7.1)
ICC 97105 × ICC 4958	Introgressing root trait QTL	1981	147 (7.4)
ICCV 10 × ICC 4958	Introgressing root trait QTL	1982	136 (6.8)
ICCV 95423 × ICC 4958	Introgressing root trait QTL	1984	124 (6.2)
JG 11 × ICC 4958	Introgressing root trait QTL	1986	119 (6.1)
DCP 92-3 × ICC 4958	Introgressing root trait QTL	1982	137 (6.9)
KAK 2 × ICC 8261	Introgressing root trait QTL	1967	40 (2.0)
ICCV 92318 (Chefe) × ICC 8261	Introgressing root trait QTL	1971	37 (1.9)
C 214 × ILC 3279	Introgressing *AB* resistance	1963	53 (2.7)
C 214 × WR 315	Introgressing *FW* resistance	1934	15 (0.8)
Phule G5 × Vishal	Introgressing *FW* resistance	1954	27 (1.4)
Phule G12 × WR 315	Introgressing *FW* resistance	1980	26 (1.3)
JG 74 × JG 14	Introgressing *FW* resistance	1959	51 (2.6)
JG 74 × WR 315	Introgressing *FW* resistance	1970	35 (1.8)
Annigeri × WR 315	Introgressing *FW* resistance	1934	34 (1.8)
Annigeri × ICCV 10	Introgressing *FW* resistance	1935	29 (1.5)
Marker-assisted recurrent selection (MARS) mapping populations			
JG 130 × ICCV 05107	Enriching drought tolerance alleles	1977	31 (1.6)
ICCV 2 × JG 11	Enriching salinity tolerance alleles and early flowering	1973	30 (1.5)
JG 11 × ICCV 04112	Enriching drought tolerance alleles	1975	27 (1.3)

SNP, single nucleotide polymorphisms.

### Genetic diversity analysis

**Figure 3:**
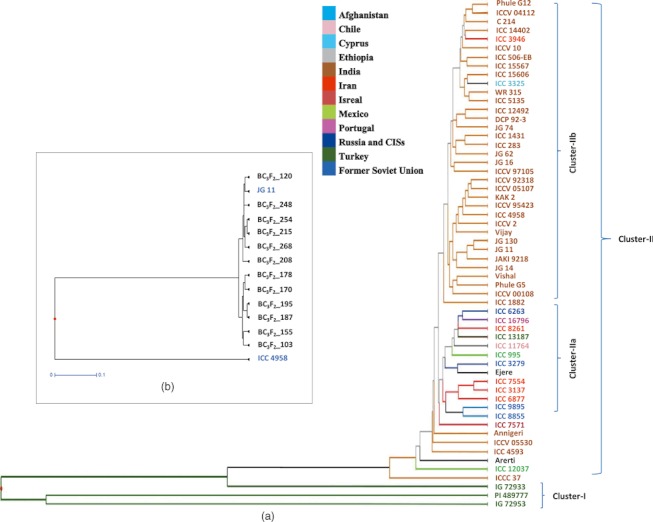
Genetic relationships in germplasm and BC_3_F_2_ lines. Hierarchical clustering of chickpea accessions was carried out based on UPGMA using DARwin. The part (a) of the figure shows phylogenetic relationships among 58 germplasm lines based on allelic data for 1341 CKAMs. All the genotypes analysed could be grouped into two main clusters (I and II). The Cluster-I comprised two wild species genotypes (*Cicer reticulatum*) and Cluster- II comprises accessions mainly of *Cicer arietinum* species coming from 11 different countries. The part (b) of the figure shows genetic dissimilarity of 12 BC_3_F_2_ lines with JG 11, the recurrent parent.

Genotyping data obtained for all 1341 polymorphic CKAMs on 58 chickpea genotypes (Table S3) were used for assessing the genetic diversity and understanding their genetic relationships. Genetic dissimilarity between different pairs of genotypes varied from 0.02 (ICC 7554 and ICC 3137) to a maximum of 0.74 (PI 48977 and IG 72933) with a mean of 0.37. On the basis of the dissimilarity data and UPGMA method, a hierarchical cluster analysis was performed on all the 58 genotypes using DARwin V5.0.128 software (Perrier *et al.*, 2003) (Figure 3). In the dendrogram, the genotypes were grouped into two discrete major clusters: the Cluster-I comprised only two wild species (*C. reticulatum*) genotypes (IG 72953 and PI 489777), and the Cluster-II comprised 56 genotypes of *C.* *arietinum* species, with an exception of one genotype IG 72933, belonging to *C. reticulatum* species, that branches off sequentially at the base of the dendrogram closer to the Cluster-I. In the Cluster-II, few landraces and cultivars from India (Annigeri, ICC 4593, ICCC 37, ICCV 05530), Ethiopia (Arerti), Mexico (ICC 12037) and Israel (ICC 7571) formed a clear outlying group, with the remaining 48 genotypes clustering into two main groups—the Cluster-IIa and the Cluster-IIb. The Cluster-IIa has 13 genotypes that mainly belong to Afghanistan (2), Chile (1), Ethiopia (1), Iran (4), Portugal (1), Turkey (1), Mexico (1) and former USSR (2). The Cluster-IIb is comprised of 35 genotypes, of which 33 belong exclusively to India, one to Iran and one to Cyprus. Within the Cluster-IIb, ICC 1882 is separated from the rest of the genotypes. Overall, the clustering pattern showed a distinctive grouping of genotypes into separate clusters based on their geographical origin and also based on species background (Figure 3a).

### Relationship of BC_3_F_2_ lines with the recurrent parent

A set of 12 BC_3_F_2_ generated after introgressing a genomic region containing QTLs for several drought tolerance–related traits in JG 11 variety after maker-assisted backcrossing (MABC) with ICC 4958 genotype were tested with all 2005 CKAMs to assess the genome recovery of JG 11 parent in the MABC lines. As a result, 108–117 markers showed similarity between the given BC_3_F_2_ line and JG 11 (Table S4). In brief, the tested BC_3_F_2_ lines showed genome recovery of JG 11 from 91% (BC_3_F_2__170, BC_3_F_2__187, BC_3_F_2__195) to 98% (BC_3_F_2__120, BC_3_F_2__248) (Figure 3b). Furthermore, comparison of the BC_3_F_2_ lines with ICC 4958 showed the presence of allele of ICC 4958 in the BC_3_F_2_ lines for 10 CKAMs (CKAM0017, CKAM1802, CKAM1444, CKAM0042, CKAM0043, CKAM1641, CKAM1963, CKAM1933, CKAM1709 and CKAM1604). These markers seem to be the potential mappable markers in the genomic region transferred from ICC 4958 to JG 11.

### Second-generation genetic map of chickpea

The reference mapping population (ICC 4958 × PI 489777) was targeted for integrating CKAMs in the genetic map of chickpea. In this context, a total of 930 CKAMs showed polymorphism between the parental genotypes. The polymorphic CKAMs include 503 Solexa/Illumina SNPs, 377 TOG-SNPs and 50 candidate gene sequencing–based SNPs. As genotyping data were already available on the reference mapping population for all 371 TOG-SNPs via GoldenGate assay, only 118 markers representing all the linkage groups were selected for genotyping via KASPar assays mainly for quality control. Therefore, genotyping data were generated on the reference mapping population for a total of 671 CKAMs (503 Solexa/Illumina SNPs, 50 candidate genes SNPs and 118 TOG-SNPs). High-quality genotyping data, however were generated for 651 CKAMs (492 Solexa/Illumina SNPs, 46 candidate genes SNPs and 112 TOG-SNPs). Analysis of genotyping data showed Mendelian segregation ratio for a total of 525 markers, and the remaining 126 (19.3%) markers exhibited segregation distortion (Table S5) owing to skewed occurrence/distribution of one of the two parental alleles or high percentage (60%) absence of allele data (Figure 2d,e,f).

**Figure 4:**
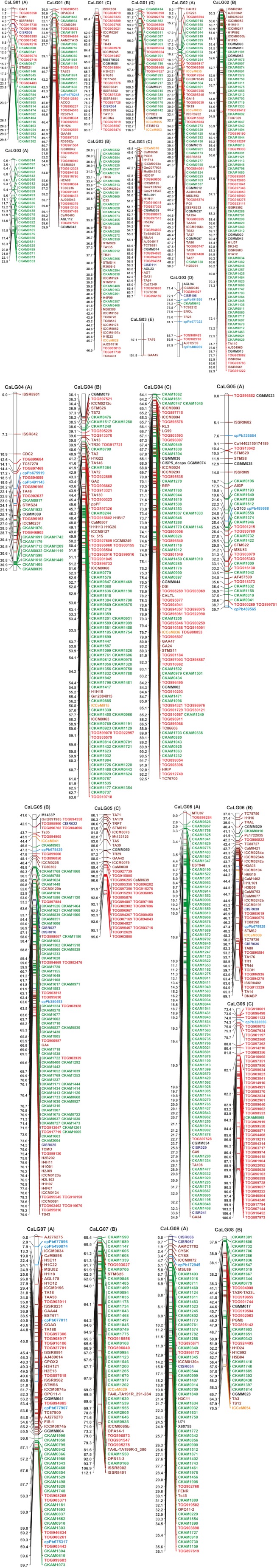
A second-generation genetic map of chickpea. The genetic map based on reference mapping population (ICC 4958 × PI 489777) is comprised of a total of 1328 marker loci including newly developed 625 CKAMs, 314 tentative orthologous genes (TOGs)-SNPs (R.V. Penmetsa, N. Carraquilla-Garcia, A.D. Farmer, R.K. Varshney, D.R. Cook, unpublished data) and 389 published marker loci in earlier studies. Eight different linkage groups are shown and designated as CaLG01 to CaLG08. For the visualization of marker names and orders, each LG has been split into 2–5 parts. For instance, four LGs, namely CaLG02, CaLG07 and CaLG08, are split into A and B parts; three LGs, namely CaLG04, CaLG05 and CaLG06, are split into A, B and C parts; the CaLG01 is divided into A, B, C and D parts; and CaLG03 is divided into A, B, C, D and E parts. Map distances (cM) are presented on the left side of the bars, and corresponding markers are listed on the right side of the bars. Each marker class is colour coded as follows: *green,* CKAMs; *red,* TOGs-SNPs; *black,* CGMMs; *dark blue,* CISRs; *golden yellow,* ICCeMs; *light blue,* DArTs; and *brown,* legacy markers. High resolution genetic map is available at http:cmap.icrisat.ac.in/cmap/sm/cp/hiremath/.

As genotyping data were available for a total of 429 TOG-SNPs via GoldenGate assay (R.V. Penmetsa, N. Carraquilla-Garcia, A.D. Farmer, R.K. Varshney, D.R. Cook, unpublished data) and high-quality genotyping data were generated for 112 TOG-SNPs from this set via KASPar assay in the study, the genotyping data for the remaining 317 TOG-SNPs generated via GoldenGate assay were added to the data set of 651 CKAMs. In addition, genotyping data were also assembled for (i) 61 genic molecular markers (GMMs) including 31 CGMMs, 15 CISRs and 15 ICCeMs (Gujaria *et al.*, 2011), and (ii) 335 legacy markers including SSRs from different sources (H-series, ICCMs, CAMs, SSRs-Frankfurt University, ISSRs), SNaPshot assays-based SNPs, CAPS, DArTs (Thudi *et al.*, 2011), and RAPDs. In summary, genotyping data were compiled for 1364 markers and used for constructing the genetic map. The most likely order of the markers was determined based on the verified position of GMMs (Gujaria *et al.*, 2011), TOG-SNPs (R.V. Penmetsa, N. Carraquilla-Garcia, A.D. Farmer, R.K. Varshney, D.R. Cook, unpublished data) and legacy markers (Nayak *et al.*, 2010; Thudi *et al.*, 2011). By using JoinMap v 4.0 program (Van Ooijen *et al.*, 2006), a total of 1328 markers were mapped onto eight linkage groups (CaLG01–CaLG08) as per the nomenclature given in Thudi *et al.* (2011). The developed genetic map spans a total of 788.6 cM distance with an average intermarker distance of 0.59 cM (http://cmap.icrisat.ac.in/cmap/sm/cp/hiremath/) (Figure 4). Details about different type of markers integrated in this map are given in Table 3. The number of markers per linkage group varied from 107 (CaLG08) to 255 (CaLG04). The total distance of individual linkage groups ranged from 70.5 (CaLG08) to 116.6 cM (CaLG01).

**Table 3:** Distribution of markers on the second-generation linkage map of chickpea

Marker type	Total markers used	Chickpea linkage group								Total markers mapped

		CaLG01	CaLG02	CaLG03	CaLG04	CaLG05	CaLG06	CaLG07	CaLG08	
CKAMs	651	52	81	57	132	90	86	59	68	625
TOG-SNPs	317	56	29	16	67	58	56	19	13	314
Published marker loci										
GMMs										
CGMMs	32	4	10	2	6	3	2	2	2	31
CISRs	15	2	–	2	–	4	4	–	3	15
ICCeMs	15	2	2	2	2	1	1	1	1	12
Legacy markers										
H-series	44	4	7	6	5	7	5	6	4	44
ICCMs	46	3	4	9	10	7	6	5	2	46
CAMs	10	1	–	1	1	2	4	1	–	10
SSRs	93	14	11	16	14	14	10	9	5	93
ISSRs	26	8	8	–	2	2	1	5	–	26
SNaPshot assay-based SNPs	79	8	8	18	12	8	8	10	7	79
CAPS	13	–	1	4	2	2	1	–	–	10
DArTs	19	1	–	3	2	5	2	5	1	19
RAPD	4	1	–	–	–	–	–	2	1	4
Total no. of markers	1364	156	161	136	255	203	186	124	107	1328
Total distance (cM)		116.6	92.94	101.8	92.5	95.6	106.6	112.1	70.5	788.6
Average intermarker distance (cM)		0.75	0.58	0.75	0.36	0.47	0.57	0.90	0.66	0.59

SNP, single nucleotide polymorphisms; SSR, simple sequence repeats; TOG, tentative orthologous genes.

Uneven distribution and clustering of markers was observed along the length of all the chickpea linkage groups in this map. Occurrence of both minor (3–5 cM) and major (>5 cM) gaps between adjacent loci was observed (Table 4). A detailed observation revealed extensive clustering of CKAMs and TOG-SNPs near the telomeric regions of CaLG03, CaLG06, CaLG07 and CaLG08 (Figure 4). In the case of CaLG01, CaLG02, CaLG04 and CaLG05, more CKAMs were clustered near the subtelomeric regions.

**Table 4:** Distribution of marker clusters on the second-generation linkage map of chickpea

Linkage group (LG)	No. of markers	Length (cM)	Intermarker distance	No. of clusters	Genetic mapping position and number of markers (in parenthesis) in clusters observed
CaLG01	156	116.6	0.75	3	23 (8), 39 (5), 61 (6)
CaLG02	161	92.94	0.58	7	17 (6), 41 (6), 53 (5), 56 (6), 57 (8), 71 (7), 72 (8)
CaLG03	136	101.8	0.75	1	35 (8)
CaLG04	255	92.5	0.36	5	52 (8), 53 (19), 54 (14), 60 (7), 30 (11)
CaLG05	203	95.6	0.47	5	53 (7), 58 (7), 69 (10), 70 (15), 91 (6)
CaLG06	186	106.6	0.57	4	3 (7), 10 (15), 19 (20), 82 (12)
CaLG07	124	112.1	0.90	2	57 (11), 64 (12)
CaLG08	107	70.5	0.66	2	8 (8), 38 (7)
Total	1328	788.6	5.04	29	
Average	166	98.58	0.63	3.6	

### Comparison of the developed genetic map with other chickpea maps

The developed genetic map with 1328 marker loci was compared with the 1291 loci genetic map (Thudi *et al.*, 2011) and 300 loci transcript map of Gujaria *et al.* (2011). The details of comparison of these maps are available at http://cmap.icrisat.ac.in/cmap/sm/cp/hiremath/. These comparisons reflect a greater congruency in terms of grouping of markers into specific linkage groups. A few exceptions were also observed. For instance, TA4L-TA199R-3_300 and TA4L-TA191R_291-284 loci were mapped on LG04 by Thudi *et al.* (2011) and on LG06 by Gujaria *et al.* (2011); these loci have been assigned to CaLG07 in the present map. Similarly, the marker loci TA5L-TS38R-1_470 and TA5L-TS129R_208 that were present on LG05 and LG08 of genetic maps developed by Thudi *et al.* (2011) and Gujaria *et al.* (2011), respectively, could not be assigned to any linkage group in this genetic map. Apart from these shifts in marker locations, no other discrepancy was observed.

### Genome relationships of chickpea with closely related legume species

**Figure 5:**
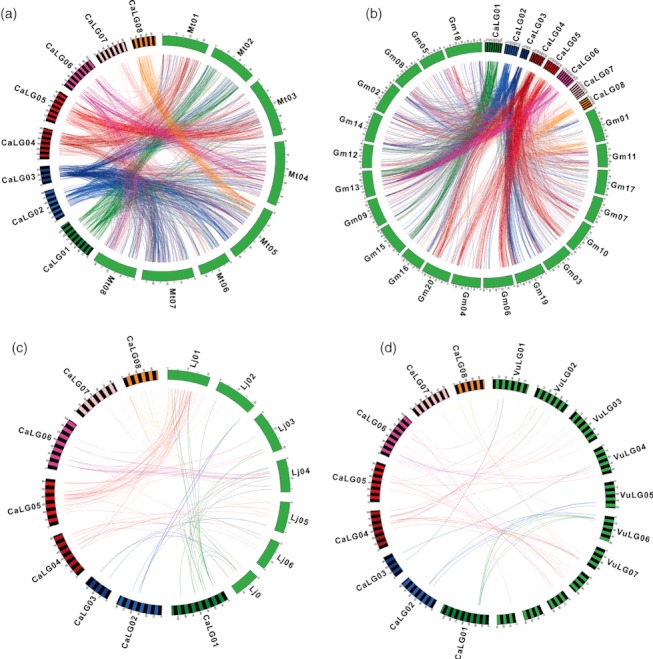
Genome relationships of chickpea with closely related legume species. Homologous relationship of chickpea genome with four legume species, that is, *Medicago truncatula* (a), soybean (b), *Lotus japonicus* (c) and cowpea (d), has been shown by comparing sequence data of 1064 mapped markers of chickpea with genome sequence of *Medicago* (Mt 3.5), *L. japonicus* (Lj 2.5 pseudomolecules), soybean (Glyma1 genome assembly) and cowpea genetic map (Muchero *et al.*, 2009). Maximum similarity was observed with *Medicago* (1558), followed with soybean genome (1798), *Lotus* (438) and least with cowpea (55). The percentage of matches in each species is in congruence with their phylogenetic distances.

We combined both the genetic map position information for chickpea loci and genome sequence information of closely related species of different clades to evaluate the degree of synteny between genomes of chickpea and other related legume species. A set of 1064 of 1328 mapped loci for which both genetic map positions and sequence information were available were compared with genome assemblies of *Medicago truncatula* (Mt 3.5), *Lotus japonicus* (Lj 2.5 pseudomolecules), soybean (*Glycine max*) (Glyma1) and the genetic map of cowpea (*Vigna unguiculata*, Muchero *et al.*, 2009) (Figure 5).

In the case of chickpea and *Medicago*, 555 unique chickpea loci showed significant matches with 1558 genomic regions on *Medicago* chromosome (Table 5). Most of the chickpea loci have ≥2 matches in *Medicago.* About 111 chickpea loci from CaLG01 showed similarity with Mtchr02 genomic regions. Similarly, loci from CaLG02 showed maximum matches to Mtchr05, followed by CaLG03 with Mtchr07, CaLG04 with Mtchr01, CaLG05 with MtChr03, CaLG06 with Mtchr04, CaLG07 with MtChr04, and CaLG08 with MtChr05. In brief, each linkage group of chickpea showed considerable synteny with one or more chromosomes of *Medicago*, although internal duplication of DNA sequences/blocks was not observed (Figure 5a).

**Table 5:** Mapping of chickpea marker loci on *Medicago* chromosomes

Chickpea linkage groups	Number of chickpea unique loci	*Medicago truncatula* chromosomes									
	
		MtChr01	MtChr02	MtChr03	MtChr04	MtChr05	MtChr06	MtChr07	MtChr08	MtChr0	Total
CaLG01	69	9	**111**	18	16	22	9	19	8	15	227
CaLG02	61	7	9	10	14	**90**	23	12	8	7	180
CaLG03	62	12	25	30	20	41	15	**99**	26	12	280
CaLG04	95	**104**	1	7	24	12	11	9	10	19	197
CaLG05	93	11	3	**129**	13	14	5	9	13	19	216
CaLG06	76	6	5	8	87	39	11	11	**51**	9	227
CaLG07	46	1	7	5	**54**	8	3	2	11	1	92
CaLG08	53	4	2	3	4	**99**	6	14	1	6	139
Total	555	154	163	210	232	325	83	175	128	88	1558

*The numbers shown in bold represent the highest matches between chickpea and *Medicago*.

In the comparison of chickpea with soybean, 494 chickpea unique loci matched 1798 short stretches distributed on different chromosomes of soybean (Glyma1 assembly) (Figure 5b, Table S6). Each chickpea marker locus showed similarity to approximately 3–4 regions on Glyma1. This reflects the number of matches one would expect to see based on the one round of whole genome duplication in soybean. Only 267 unique chickpea loci matched with 438 regions on *Lotus* (Table S7, Figure 5c). In the case of cowpea in which genetic map was used for the comparison, least matches were observed between chickpea and cowpea genomes. Only 50 unique chickpea loci showed synteny with 55 loci of cowpea map (Table S8, Figure 5d).

## Discussion

### Cost-effective KASPar assays for SNP genotyping

Until recently, SSR markers were the commonly used markers for chickpea genetics research and breeding applications (Upadhyaya *et al.*, 2011). Nevertheless, in some cases, genetic maps have also been developed using DArTs (Thudi *et al.*, 2011), CISRs (Gujaria *et al.*, 2011) and SNPs/CAPs (Choudhary *et al.*, 2012; Gujaria *et al.*, 2011; Nayak *et al.*, 2010). With the availability of whole genome or EST sequences in many crop species, the use of SNP markers has been proven attractive for high-throughput use in molecular breeding (Rafalski, 2002; Varshney, 2010). High-throughput SNP genotyping platforms such as Illumina’s GoldenGate or Infinium assays are being used for large-scale SNP genotyping. While the high-throughput SNP genotyping platforms are very useful for rapid genotyping of mapping population or germplasm collections, they are not generally economical for projects such as *in silico* SNP validation, gene-specific SNP assays, marker saturation in the regions of interest and marker application projects that utilizes defined set/panel of smaller number of SNP markers on varying number of genotypes. In such cases, SNP genotyping technologies such as arrayed primer extension reaction (APEX) (Podder *et al.*, 2008), dynamic allele-specific hybridization (DASH) (Podder *et al.*, 2008), molecular beacons (Mhlanga and Malmberg, 2001), primer extension followed by MALDI-TOF (alternative to Sequenom’s assays) (Sauer *et al.*, 2000) and KASPar assay (http://www.kbioscience.co.uk/reagents/KASP.html) have been developed. While choosing a particular SNP genotyping platform, several features such as the reproducibility, accuracy, capability of multiplexing, the level of throughput, time consumption and cost (considering both the equipments required and the cost per genotype) need to be considered. As molecular breeding applications, generally, require screening of large populations with a few markers, this study developed cost-effective KASPar marker assays for SNP genotyping in chickpea.

A total of 2486 SNPs were assembled from different sources for developing KASPar assays. KASPar assays developed for chickpea have been referred as CKAMs. Genotyping of these 2486 CKAMs on a panel of 70 genotypes provided a validated set of 2005 CKAMs. This includes KASPar assays for 539 TOG-SNPs that were initially assayed on GoldenGate assays. Conversion of these TOG-SNPs into KASPar assay will facilitate use of TOG-SNPs in chickpea genetics and breeding application.

To compare the success rate of converting putative SNPs into successful and informative KASPar assays, amplification and polymorphism statistics were checked across the four sets of SNPs. The set of markers that gave higher rate of failures were those SNPs identified from alignments of Sanger ESTs (172 SNP markers, i.e. 60% of a total of 286). The possible reasons could be attributed primarily to (i) SNPs were mined from the ESTs with sequencing artefacts, (ii) frequency of one of two alleles for a given SNP is very low in the EST data set, and (iii) all the genotypes for which EST-based mining approach provided SNPs were not included in the genotype panel used in the current study (Varshney *et al.*, 2009). The remaining number of markers that could not be validated include 222 (15.7% of total of 1409) from Alpheus pipeline predicted SNPs, 65 SNPs (10.7% out of 604) from TOG-SNPs and 22 SNPs (11.7% out of 187) from allele resequencing data. Overall, the KASPar assay has shown 81% validation success rate in our study. Comparison of costs and time involved in genotyping the SNPs via KASPar assays and GoldenGate assays for the same set of SNPs in this study, showed superiority of KASPar assays over GoldenGate assays, especially when limited number of SNPs (<500) are genotyped with <100 lines.

The PIC values of validated CKAMs varied from 0.02 to 0.50 with an average of 0.12. Low range of PIC value of CKAMs is not unexpected as genetic variation in the chickpea gene pool is limited (Nayak *et al.*, 2010; Thudi *et al.*, 2011). Also, this study identifies polymorphic markers (15–930) for different mapping populations segregating for drought, salinity, *Fusarium wilt*, Ascochyta blight, etc. It, therefore, provides opportunities for mapping resistance to biotic and tolerance to abiotic stresses in chickpea.

### Diversity analysis and molecular breeding applications

This study demonstrates the suitability of KASPar assays for SNP genotyping for understanding the relationships in the germplasm collection as well as for molecular breeding applications. Despite using a wide diverse collection of genotypes with all 2005 CKAMs, an overall success rate of 81% was achieved. The genetic dissimilarity analysis of the germplasm accessions determines relationships of accessions with each other. The dendrogram developed based on genetic dissimilarity coefficient depicted clear clustering of chickpea accessions into two main clusters as per their geographical origin and species type of all 58 accessions (55 accessions of *C. arietinum* species and three accessions of *C. reticulatum* species) analysed. Two accessions of *C. reticulatum* are resolved as a separate group; however, IG 72933, a *C. reticulatum,* was found closer to *C. arietinum*. Similar results were observed in an earlier genetic diversity study using 513 SSR markers in which the IG 72933 genotype showed 40% similarity with the *C. arietinum* genotypes (Gudipati, 2007). The Cluster-II contained more geographically divergent material of the *C. arietinum* species. As expected, accessions of all Indian origin formed a separate clade, and the remaining accessions from other countries were grouped into another clade (IIa). Overall, these results are in general congruence with earlier studies and indicate that the cluster topology is reliable.

The study also demonstrates the utility of CKAMs for assessing the genome recovery of BC_3_F_2_ lines. This study identified five lines (BC_3_F_2__120, BC_3_F_2__170, BC_3_F_2__187, BC_3_F_2__195 and BC_3_F_2__268) with > 95% genome recovery of JG 11 in MABC experiments. These lines may be used for multi-location field trials for evaluating agronomic performance as well as for developing the near isogenic lines (NILs) for fine mapping the QTLs.

### Second-generation genetic map of chickpea with more anchoring points with other legume genomes

As expected, the number of polymorphic markers observed between interspecific mapping populations is higher than intraspecific mapping populations. For instance, maximum number of polymorphic markers is 930 (ICC 4958 × PI 489777) in interspecific crosses as compared with 159 (Arerti × ICC 4958) in intraspecific crosses. As ICC 4958 × PI 489777 population is a reference mapping population, genotyping data were generated for the polymorphic CKAMs. Although genotyping data were earlier generated for TOG-SNPs on the mapping population via GoldenGate assays (R.V. Penmetsa, N. Carraquilla-Garcia, A.D. Farmer, R.K. Varshney, D.R. Cook, unpublished data), a set of 118 TOG-SNPs distributed on all eight LGs was also targeted for generating genotyping data via KASPar assays for quality control purpose. Comparison of high-quality data for 112 markers generated via KASPar assay with that of GoldenGate assay showed no discrepancy. After assembling genotyping data for 539 remaining CKAMs, 317 TOGs and 396 marker loci from other sources (Gujaria *et al.*, 2011; Nayak *et al.*, 2010; R.V. Penmetsa, N. Carraquilla-Garcia, A.D. Farmer, R.K. Varshney, D.R. Cook, unpublished data; Thudi *et al.*, 2011), genotyping data for a total of 1364 marker loci were considered for mapping. As a result, a comprehensive genetic map comprising 1328 marker loci including 939 new marker loci (625 CKAMs, 314 TOGs-SNPs) and 389 already published mapped marker loci was developed. The second-generation genetic map has a coverage of 788.6 cM genetic distance. On an average, each of the linkage group has 166 markers with an average distance of 98.6 cM. This map has probably the highest number of gene-based SNP markers (1088) mapped in chickpea so far. Earlier to this map, Gujaria *et al.* (2011) developed a transcript map with 126 gene-based markers and Choudhary *et al.* (2012) developed a genetic map with 406 marker loci including 177 gene-based markers. This map has approximately eightfold gene-based markers as compared with the above-mentioned studies. Another important feature with this genetic map is the availability of cost-effective KASPar assays for the mapped gene-based markers that can be used in any number as well as on a variable number of lines. The quality and accuracy of the second-generation genetic map was evaluated by comparing it with several genetic maps developed in earlier studies (Gujaria *et al.*, 2011; Nayak *et al.*, 2010; Thudi *et al.*, 2011; Winter *et al.*, 1999).

Clustering of two or more markers is a commonly occurring phenomenon observed in several earlier genetic maps of chickpea (Nayak *et al.*, 2010; Thudi *et al.*, 2011; Winter *et al.*, 1999). Only CKAMs and TOG-based SNPs were clustered, which constitute a large proportion of mapped markers [i.e. 625 CKAMs and 314 TOG-SNPs (939, 71%) of 1328] compared with other marker types. This clustering may be attributed mainly to random selection of markers from the closely spaced regions of the genome that have undergone comparatively less number of recombination events.

As a complement to the gene-based linkage map developed in this study, we compared the sequences of these mapped loci with genome assemblies/genetic maps of four legume species (*Medicago*, *Lotus*, cowpea and soybean). Through the comparative analysis, high conservation of synteny was observed between chickpea and *Medicago*, whereas lowest level of synteny conservation was observed between chickpea and cowpea. Apparently, during the time of analysis genome sequence information was not available for cowpea; hence, the analysis was carried out by comparing with high-density linkage map developed by Muchero *et al.* (2009) available then. As a result, least similarity was identified between chickpea and cowpea, although chickpea is phylogenetically closer to cowpea than it is to soybean, which shares the same common ancestor relative to the ancestor of chickpea, *Medicago* and *Lotus* (Wojsciechowski *et al.*, 2004). In all the other cases, high level of similarity was observed (>70%, 1E-05) between sequences of chickpea, and those of compared legumes, however, are often punctuated or interrupted by chromosomal rearrangements, thereby resulting in disruption of the linear order of the genes. Subsequently, these variations (insertion, deletion, duplication or rearrangements) form the basis for evolution of diverse genomes. One or more chickpea loci match to a single locus on *Medicago* chromosome, and similar pattern was observed for the remaining three legume genomes with chickpea. This may reflect segmental duplication events of chromosomal stretches, or the mapped loci may correspond to paralogous genes or same gene family members. Recent analysis of *Medicago* genome has revealed that higher rates of mutations and chromosomal rearrangements are known to have occurred after the whole genome duplication event as compared with other model legumes such as *Glycine max* and *L. japonicus* (Young *et al.*, 2011).

A number of chickpea unique loci matching to different chromosomal regions on Mt 3.5, Glyma1, Lj 2.5 and cowpea genetic map were identified. Of the 69 chickpea unique loci that mapped on 227 regions distributed over eight chromosomes of *Medicago*, approximately 49% (i.e. 111 of 227) matched to the MtChr02 and the remaining 116 were similar to those on other chromosomes. Only 53 loci are in linear order with Mtchr02 chromosomal regions, and the remaining are in nonlinear positions. These findings support the earlier reports by Choi *et al.* (2004), Nayak *et al.* (2010) and Zhu *et al.* (2005) that one to one synteny does not hold true between chickpea and the compared legume species, and the synteny is restricted only to small genetic or genomic intervals (Young *et al.*, 2011). Our comparative results showed that regions of CaLG02 and CaLG08 are strongly similar to Mt05, which in turn shows high similarity to regions on Gm01, Gm02 and Gm11, which are consistent with the findings of Young *et al.* (2011).

## Conclusions

The study reports compilation of a large number of SNPs and their conversion into cost-effective KASPar assays. A set of 2005 KASPar assays have been developed for accelerating chickpea genetics research and breeding applications. Together with these markers and recently developed SSR markers from genomic libraries (Nayak *et al.*, 2010) and BAC-end sequences (Thudi *et al.*, 2011), DArT markers (Thudi *et al.*, 2011), CISR- and CAPS-based CGMMs, >10 000 markers have become available in chickpea. The available marker resource should be able to tackle the issue of narrow genetic diversity in the gene pool as it is now possible to identify reasonable number of polymorphic markers in any given combination of cross. Genetic structure information gained on 58 chickpea accessions may be useful in finding suitable parental combinations for developing the new mapping populations segregating for different traits of interest to chickpea breeders. Furthermore, a number of polymorphic markers were identified in many existing mapping populations that can be used for developing genetic maps and mapping of different agronomic traits. Many polymorphic markers were found to be common in many mapping populations, revealing their usefulness in providing bridging markers and for comparing different chickpea maps. Developed genetic map is the most enriched genetic map for gene-based markers. This map should be useful not only in comparing different chickpea genetic maps, but also in anchoring the physical map, currently underway, as well as establishing more anchor points among genomes of chickpea and other legume species.

## Experimental procedure

### Plant material and DNA extraction

A set of 70 different chickpea genotypes was used for validation of SNPs using KASPar assays. Details of these genotypes are given in Table 2 and Table S2. Furthermore, a set of 131 recombinant inbred lines (RILs) derived from the cross between ICC 4958 (*C.* *arietinum)* and PI 489777 (*C.* *reticulatum)* was used for genetic mapping.

Total genomic DNA of all the accessions was extracted from leaves of two-week-old seedlings using high-throughput mini DNA extraction protocol as mentioned in Cuc *et al.* (2008). The quality and quantity of extracted DNAs were assessed on 0.8% agarose gel. The DNA was normalized to 5 ng/μL for genotyping.

### RNA Sequencing by Solexa/Illumina

Five different chickpea genotypes, viz. ICC 4958, ICC 1882, PI 489777, ICC 506 and ICCC 37, which are parents of different mapping populations, were selected for RNA sequencing. Roots of 22-day-old seedlings of ICC 4958 and ICC 1882 were subjected to drought stresses, and subsequently total RNA was extracted from both genotypes (Hiremath *et al.*, 2011). About 22-day-old leaves of ICC 506 and ICCC 37 were infested with larvae of *Helicoverpa armigera* for a period of 5 days under green house conditions (temperature of 28 ± 5 °C and relative humidity of >65%). After a brief infestation period, leaf samples from both genotypes were harvested for total RNA extraction. Total RNA was also extracted from 22-day-old root tissues of PI 489777, a wild species genotype. Subsequently, the total RNA samples of all the genotypes were sent for Solexa/Illumina sequencing at National Center for Genome Research (NCGR), USA.

### Development and analysis of KASPar assays

For developing the KASPar assays, 50 bp upstream and 50 bp downstream flanking sequences around the variant position (SNP) were selected (Table S1). Subsequently, KASPar assays for the targeted SNPs were carried out at KBioscience, UK. Complete details on principle and procedure of the assay are available at http://www.kbioscience.co.uk/reagents/KASP_manual.pdf and http://www.kbioscience.co.uk/download/KASP.swf. On the basis of the fluorescence obtained, allele call data are viewed graphically as a scatter plot for each marker assayed using the SNPViewer. The consistency between the predicted SNP and assayed ones was checked for each SNP marker.

### Evaluation of polymorphism in chickpea accessions

The PIC refers to the value of a marker for detecting polymorphism within a given germplasm, depending on the number of detectable alleles and the distribution of their frequency. In this study, the PIC value of markers was calculated using the following formula (Anderson *et al.*, 1993):


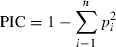


Where ‘*n*’ denotes the total number of alleles and ‘*p*’ refers to the frequency of the ‘*i*’th allele at a genetic locus in different genotypes.

### Genetic diversity analysis

To evaluate the relationship between chickpea germplasm accessions, SNP allele call data obtained for polymorphic markers were used for calculating both pair-wise genetic distance and per cent dissimilarity matrix to construct a dendrogram using DARwin V5.0.128 software (http://darwin.cirad.fr/darwin/Home.php, Perrier *et al.*, 2003). Cluster analysis was carried out using the UPGMA method.

### Genetic mapping

Genotyping data obtained using KASPar assays (CKAMs) were compiled with the marker data for TOGs-SNPs (R.V. Penmetsa, N. Carraquilla-Garcia, A.D. Farmer, R.K. Varshney, D.R. Cook, unpublished data) and selected markers from all 8 linkage groups mapped in earlier studies (Gujaria *et al.*, 2011; Nayak *et al.*, 2010; Thudi *et al.*, 2011). Segregation data for CKAMs were tested for goodness of fit to the expected Mendelian ratio of 1:1 using chi-square (χ^2^) analysis (*P* < 0.05). All markers were primarily divided into linkage groups using the ‘group’ command of mapmaker/exp 3.0 program (Lander *et al.*, 1987). However, to construct high-quality genetic map, those markers grouped by mapmaker were mapped using JoinMap 4 program (Stam, 1993; Van Ooijen, 2006; http://www.kyazma.nl/index.php/mc.JoinMap/). ‘Kosambi’ mapping function was used to calculate centimorgan (cM) distances. LOD values ranging from 3 to 7 were considered for grouping and mapping. MapChart (2.1v) was used for drawing maps (Voorips, 2002, http://www.biometris.wur.nl/uk/Software/MapChart/).

### Comparative mapping between chickpea and closer legumes

Sequences data for mapped chickpea marker loci were queried using BLAST against genomes of *M. truncatula* (Mt 3.5), *L. japonicus* (Lj 2.5 pseudomolecules), soybean (Glyma1 genome assembly) and cowpea genetic map (Muchero *et al.*, 2009). All the databases mentioned are available at http://comparative-legumes.org/. Hits matching a minimum of 70% sequence identity were retained for comparative study. Identification of homologous blocks was performed using i-ADHoRe v2.1 (Vandepoele *et al.*, 2002). For the purpose of developing Circos images, cM distances on the chickpea linkage groups were scaled up by a factor of 250 000 to match similar base pair lengths of the chromosomes of other legumes’ genomes. Visualization of blocks was performed with Circos26. Scales along the outer edge of the chickpea linkage groups show actual cM distances, while the scale along the outer edge of the *Medicago* chromosomes are in Mb.

## Supporting information

**Table S1** Details on 2486 selected SNPs alongwith their SNP variants, 50 bp upstream and downstream flanking sequence, marker ID and PIC values.

**Table S2** Details on 70 chickpea genotypes used for validation of 2486 CKAMs.

**Table S3** Details of genotyping data for 1341 CKAMs on 58 chickpea genotypes.

**Table S4** Details of genotyping data for 119 CKAMs on 12 BC_3_F_2_ lines along with parental genotypes ICC 4958 and JG 11.

**Table S5** Details of genotyping data on 131 RILs for 651 CKAMs and their respective Chi-square (χ^2^) test values.

**Table S6** Detailed results on comparison of mapped marker loci of chickpea with soybean (*Glycine max*) genome.

**Table S7** Detailed results on comparison of mapped marker loci of chickpea with *Lotus japonicus* genome.

**Table S8** Detailed results on comparison of mapped marker loci of chickpea with cowpea genetic map.
